# Evidence for a shared cognitive mechanism underlying relative rhythmic and melodic perception

**DOI:** 10.3389/fpsyg.2024.1512262

**Published:** 2025-01-15

**Authors:** Jeroen van der Aa, W. Tecumseh Fitch

**Affiliations:** Department of Behavioral and Cognitive Biology, Vienna CogSciHub, University of Vienna, Vienna, Austria

**Keywords:** rhythm, melody, music, human, auditory perception, relative information

## Abstract

Musical melodies and rhythms are typically perceived in a relative manner: two melodies are considered “the same” even if one is shifted up or down in frequency, as long as the relationships among the notes are preserved. Similar principles apply to rhythms, which can be slowed down or sped up proportionally in time and still be considered the same pattern. We investigated whether humans perceiving rhythms and melodies may rely upon the same or similar mechanisms to achieve this relative perception. We looked at the effects of changing relative information on both rhythm and melody perception using a same-different paradigm. Our manipulations changed stimulus contour and/or added a referent in the form of either a metrical pulse (bass-drum beat) for rhythm stimuli, or a melodic drone for melody stimuli. We found that these manipulations had similar effects on performance across rhythmic and melodic stimuli. To our knowledge, this is the first study showing that the addition of a drone note has significant effects on melody perception, warranting further investigation. Overall, our results are consistent with the hypothesis that relative perception of rhythm and melody rely upon shared relative perception mechanisms, alongside domain specific mechanisms. Further work is needed to explore the specific nature of this relationship and to pinpoint the cognitive and neural mechanisms involved.

## Introduction

Relative, rather than absolute information processing seems to play a prominent role in human music perception and production. One example of this is relative pitch, which allows us to recognise the melody of familiar melodies, such as “Happy Birthday”, regardless of which note, i.e., absolute pitch, the melody begins on. Proposals of possible human musical universals include both relative pitch and components dependent on relative feature perception in the rhythmic domain, such as isometric patterns, and the divisive organisation of temporal structure (e.g., an isochronous beat organised into metre; Brown and Jordania, 2011; Savage et al., 2015). More specifically, the cognitive and biological mechanisms underlying the perception and production of music which lead to some proposed musical universals—components of human cognition generally referred to as musicality (Honing and Ploeger, 2012; Honing et al., 2015)—are rooted in relative information. For temporal structure, this involves metrical encoding of rhythm, beat perception and synchronisation, and for pitch it involves the relative tonal encoding of frequency (Peretz and Coltheart, 2003; Trehub, 2003). Given the importance of relative processing in both pitch and rhythmic domains, a better understanding of relative feature perception across these two components of music perception could lead to fundamental insights into music and musicality.

Relative pitch refers to the phenomenon that two melodies containing the same inter-note fundamental frequency ratios are perceived as being the “same”, even if one melody is pitch shifted relative to the other (Dowling and Fujitani, 1971). Humans are particularly well attuned to perception of relative pitch (Peretz and Hyde, 2003; Peretz and Vuvan, 2017), and this capability does not require formal training, developing spontaneously over human ontogeny (Hulse et al., 1992; Plantinga and Trainor, 2005; Saffran, 2003). This in contrast to absolute pitch, the ability to identify a musical note without external reference, which is relatively rare and requires extensive musical training early in life (Bachem, 1955; Di Stefano and Spence, 2024). Interestingly, it seems that increased proficiency in absolute pitch perception comes at a cost regarding relative pitch perception capabilities (Miyazaki and Rakowski, 2002; Moulton, 2014).

Similar to relative pitch, information in the rhythmic domain is also encoded relatively, where rhythmic patterns are determined by the duration interval ratios between event onsets. A change in tempo, i.e., a scaling of all the intervals without changing the ratios, leaves the rhythmic pattern intact. The relativity of rhythm is reinforced by metre, which creates a grid of expectation with higher salience for some locations in time than others (Fitch, 2013; Longuet-Higgins, 1979). This can lead to a divisive organisation of the durational and/or rhythmic structure, most commonly divisions of 2 and/or 3, applicable to both metre and the rhythmic patterns themselves (Longuet-Higgins and Lee, 1984; Savage et al., 2015). This relative perception of auditory events in time is distinct from its absolute counterpart, duration estimation of a single interval (Repp and Su, 2013; Teki et al., 2011, 2012).

A recent study with individuals with autism spectrum disorder (ASD) found reduced imitation capabilities specific to absolute features both in pitch and timing, with unimpaired imitation of relative features (Wang et al., 2021). Moreover, these differences were found for both song and speech stimuli, suggesting domain general absolute and relative capabilities, at least across the auditory domain. Similarly, in a majority of individuals with congenital amusia, commonly known as tone-deafness, beat perception and production capabilities were found to be impaired, similar to their relative pitch capabilities (Lagrois and Peretz, 2019). In non-human animals, both relative pitch (see Hoeschele, 2017 for an overview) and relativity in timing or rhythm (e.g., Cook et al., 2013; Patel, 2021; Patel et al., 2009; Schachner et al., 2009) seem to be the exception, not the norm. Interestingly, in non-human primates, absolute feature perception and production tasks yield human-like performance, but relative capabilities are at best limited (Brosch et al., 2004; Hattori et al., 2013; Izumi, 2001; Merchant and Honing, 2014; Selezneva et al., 2013; Zarco et al., 2009). Given the evidence above, in this experiment we aim to explore whether relative feature processing is shared between rhythm and melody perception in humans.

To investigate this potential relationship, we conducted a perceptual experiment using a same/different paradigm: Participants were presented with two stimuli and asked to identify whether they differed or were the same, with separate trials for melodic and rhythmic stimuli. Hypothesising that components of relative perception are shared between rhythm and melody, we predicted that manipulations of relative features should have similar effects in both domains.

Manipulations used in this experiment include the effects of changing contour and the addition of a referent on perception of both rhythm and melody. On some trials the second stimulus presented contained deviations that either conformed or did not conform to the contour of the original sound. In addition, during half of the trials both stimuli contained a referent. The referent contained additional information that aligned with, and thus might perceptually reinforce, the relative aspects of the melody or rhythm of the sound. Based on previous work, we predicted that changes in contour will be easier to detect, and thus lead to better performance in both rhythm trials and melody trials (Dowling and Fujitani, 1971; Weiss and Peretz, 2024). This would provide support for the idea that contour is a salient perceptual feature not only in the pitch domain (McDermott et al., 2008), but potentially also in the rhythmic domain. Similarly, we predicted that the referent would make the task easier for participants for both the rhythm and melody conditions, by providing a perceptual anchor for relative information processing and thereby making perception and subsequent judgment easier and more consistent. Finally, if relative perception in the two domains relies on some shared cognitive resources, we predicted that variability in individual performance will be similar across domains, therefore leading to a positive correlation between performance in the rhythm and melody conditions.

## Materials and methods

### Participants

Sixty participants (29F, 31M; 27, 25 ± 7, 27 years) were recruited for online participation using Labvanced crowdsourcing (www.labvanced.com). An additional eleven participants (6F, 5M, 32 ± 7, 51 years) were invited into a controlled laboratory setting at the University of Vienna. Recruitment for on-site participants was done using the Vienna Cognitive Science Hub: Study Participant Platform, which utilises the hroot software (Bock et al., 2014). Musical training was no selection criteria and is thus random with regards to the recruitment pool. Of the online population, 22 out of 60 participants were considered musically trained (see details below), as were 4 of the 11 on-site participants. All participants reported to not possess any visual, hearing or any other neurological impairments. See Table 1 for an overview of demographic and technical information.

**Table 1:** Overview of participant demographic and technical information.

	**Online**	**Laboratory**
Sex	Female: 29	Female: 6
	Male: 31	Male: 5
Age (± s.d.; min–max)	27.25 (7.27; 19–50)	32 (7.51; 23–46)
Continent of birth	Africa: 21	Asia: 2
	Asia: 1	Europe: 8
	Europe: 14	Latin America: 1
	Latin America: 23	
	North America: 1	
Musical experience score (±s.d.; min–max)	5.35 (2.82; 1–12)	4.45 (2.46; 2–10)
Musical training	22/60	4/11
Computer	Laptop: 39	Desktop PC: 11
	Desktop PC: 21	
Playback device	Headphones: 27	Headphones: 11
	Earphones: 15	
	External speakers: 6	
	Internal speakers: 12	

All participants joined the experiment voluntarily, provided written consent, and received monetary compensation for completed participation. Experiments took place between December 2022 and March 2023. This experiment was approved by the Ethics Committee of the University of Vienna under reference number 00808.

### Stimuli

All stimuli were created with a custom Python (3.8.12) JupyterLab (3.0.14) notebook using the music21 package (7.1.0, pypi.org/project/music21/). Music21 was used to generate MIDI files, which were converted to WAV using fluidsynth (2.3.0, www.fluidsynth.org). Stimuli were generated in sets based on a semi-random base pattern, the target. All patterns were in 4/4 metre, with 160 beats per minute. The playback instrument for the rhythm target pattern was a snare drum rim hit (GM Midi percussion instrument 37) and, for the melody target pattern, a piano (GM Midi instrument 1).

#### Target patterns

Rhythm target patterns were generated by randomly selecting an interval from 0.5, 1, 1.5, or 2 quarter notes (8th to half note intervals) in a way that limited the total stimulus duration to 3 4/4 measures. The number of intervals varied between rhythmic stimuli, and ranged from 8 to 12 intervals, i.e., 9–13 events. A rhythmic pattern always started on the first beat of the first measure and the rhythm pattern only consists of snare drum events. Melody target patterns were generated by randomly selecting a note from the 4th octave (midi notes 60–71), which functioned as the root in the major pentatonic scale from which notes were subsequently randomly selected. The range of notes consisted of a whole octave, from semitone −5 to +7 relative to the root, i.e., six possible notes from perfect 5th below up to and including the perfect 5th above the root. The first note in each pattern was always the root of the scale, and the interval range was from ±2 to ±12 semitones compared to its preceding interval. Each individual note was 0.5 quarter note duration (8th note) and the melody stimulus duration was 2 4/4 measures, i.e., 16 notes and 15 intervals. Sixteen different randomly generated targets were generated for both the melody and rhythm stimulus sets.

#### Manipulations

For all targets, four different manipulations were applied to a single event, for each possible interval position in the pattern. In order to prevent the overall rhythmic or melodic pattern of being affected beyond the intended interval, the following interval was adjusted in the opposite direction of the manipulation. As such, each manipulation affected a pair of intervals, or one single event, except for when the manipulation was applied to the last interval in the pattern. The possible manipulations were ±0.25 and ±0.125 quarter note (16th and 32nd note duration) for the rhythmic patterns and ±2 and ±3 semitones for the melodic patterns.

**Figure 1:**
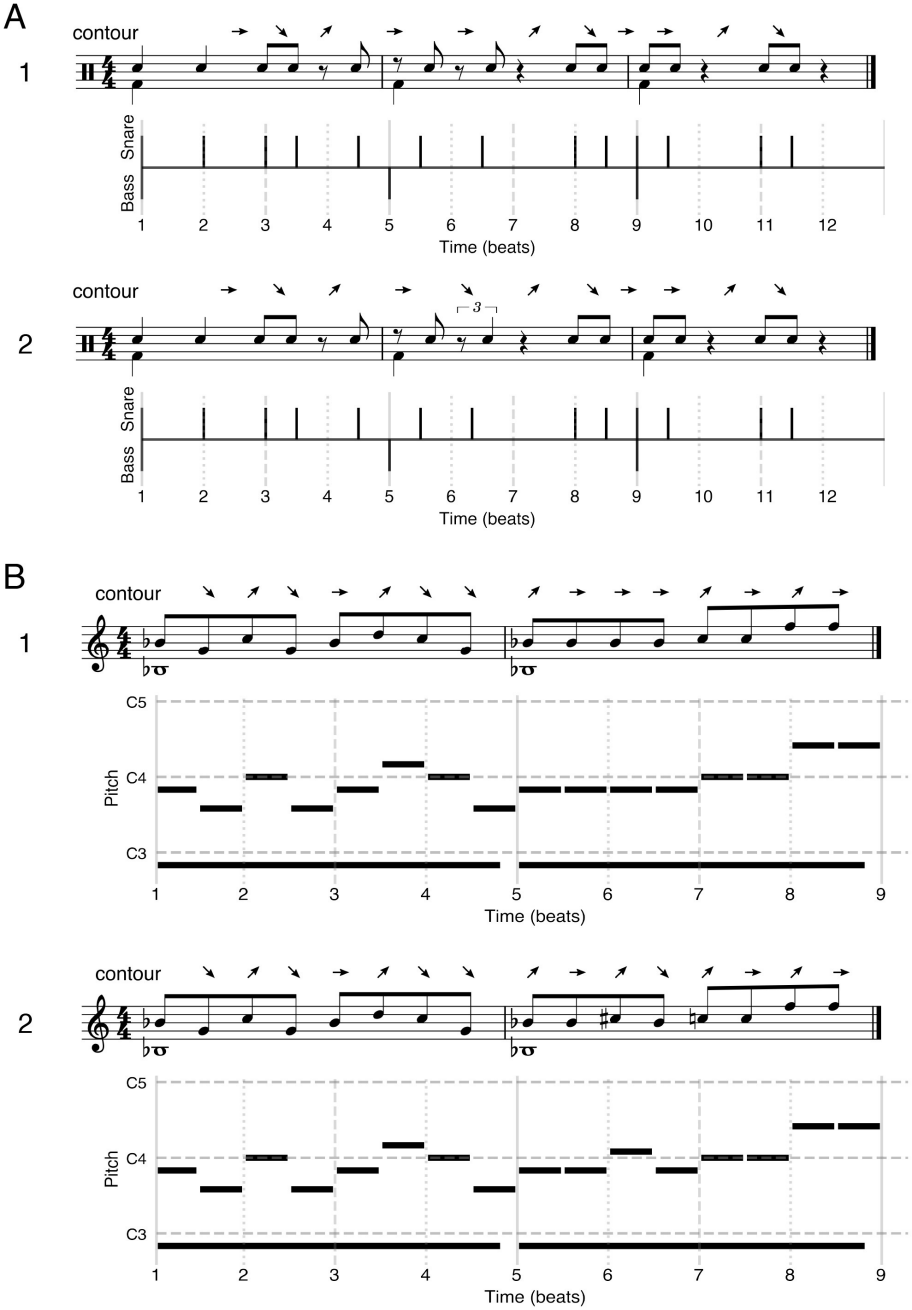
Examples of rhythm **(A)** and melody **(B)** stimuli used in the experiment, including contour scoring above them. (1) Example of a target stimulus including referent. (2) Example of a “different” stimulus including referent, with a contour change and scored as out-of-framework. All examples include musical notation **(top)** and pianoroll style **(bottom)** notation. Above each musical notation, the contour of the stimulus pattern has been notated. Arrow direction refers to shorter (↘), longer (↗), or identical (→) inter onset interval as compared to the preceding interval for the rhythmic pattern. For melody stimuli the arrow direction refers to lower (↘), higher (↗), or identical (→) pitch as compared to the previous note.

For all generated stimuli, a second version was generated where a referent was added to the pattern. For rhythm stimuli the referent consisted of a bass drum beat event (GM Midi percussion instrument 36) on the first beat of every measure. For the melody stimuli the referent consisted of a sine wave with a frequency equal to the scale root lowered by a whole octave with a duration of an entire measure (i.e., drone note, 1/20 total note duration fade in and fade out) for each measure. This referent was generated using the pydub package (0.25.1, pypi.org/project/pydub/, volume setting: −15) directly to wav file, and added to the wav version of the original pattern using the pydub overlay functionality. See Figure 1A for rhythm stimuli, and Figure 1B for melody stimuli examples.

For each manipulation per target pattern, we calculated the contour of the pattern. For the melodic patterns, we assessed whether pitch was higher, lower, or identical as compared to its preceding event, with the same logic applied to interval duration for the intervals in the snare rhythmic patterns. We then randomly selected a pattern where the contour was changed and unchanged relative to the target for inclusion in the experiment, per target, per manipulation. This led to a total of 4 manipulations × 2 contour impacts + the target = 9 stimuli. These 9 stimuli were created both with and without the referent, leading to a total of 18 × 16 (different target patterns) = 288 stimuli for both the rhythm and melody conditions.

For future analyses, we characterised the impact of the manipulation on the rhythmic and melodic expectations as suggested by the stimuli, i.e., framework. In the case of the melody stimuli, the framework is the pentatonic scale, and therefore we scored whether the changed note was either in or out of the major pentatonic scale (i.e., in/out-of-key). For the rhythm stimuli, the effect within the 4/4 metrical framework was scored. As such we calculated the syncopation of all rhythm stimuli according to the Longuet-Higgins and Lee (LHL) syncopation score as later expanded on by Fitch and Rosenfeld (2007; LHL-FR). Since rhythmic patterns without referents do not enforce a metric framework, and Fitch and Rosenfeld (2007) showed that listeners are likely to reinterpret highly syncopated rhythms as less syncopated, we calculated the lowest LFL-FR syncopation score possible for these patterns in a 4/4 metre. This was done by calculating the score for each pattern iteratively shifted by a 16th note for one measure, saving only the lowest score. The difference in syncopation between the target and manipulated pattern was used as a measure of framework impact, where a difference of 4 or larger was considered out-of-framework. This difference equates to a strongly syncopated effect of this single event change (e.g., a downbeat being offset by a 32nd note). This led to a balanced split of manipulations being scored as in-framework and out-of-framework for the rhythm stimuli, where the syncopation difference range was from 0 to 15.

### Experimental procedure

The experiment was built and run using the Labvanced experimental platform (labvanced.com), accessed through a web browser using a keyboard, mouse and an audio playback device. In lab participants conducted the experiment on an Apple iMac (21.5”; Retina 4K; 2017) in Chrome (version 109.0.0.0 or higher), using over-ear headphones (AKG K-52; 18–20.000 Hz). After providing informed consent and filling out a questionnaire on demographic information and musical experience, participants were provided with a description of the experimental procedure. The musical experience questions were modelled after the Gold MSI musical experience subset (Müllensiefen et al., 2014), focussing on instrumental (including singing), dancing and listening experience. Participants were considered musically trained for future analyses if they received at least a year of instrumental and/or dancing training. This criterion was chosen after later analysis found it to be a good predictor for the combined musical experience score (Supplementary Figure S1), with additional years of training not being a better predictor. After filling out the questionnaire, participants were asked to set their devices' volume “to an audible and comfortable level.” This was facilitated by a melodic and a rhythmic stimulus that could be played as often as desired and were not used in the experiment. After this participants completed two practise trials, using non-experimental musical stimuli which were generated using Music21 using the same instruments as the experimental stimuli.

A trial could be started by participants whenever ready by clicking a centred button using a mouse, after which they were played two stimuli of a single category (rhythm or melody), separated by a 2 s silence. Stimuli were presented once, without the possibility to re-listen or go back. The first stimulus was always an unmanipulated version, the second could be a manipulated version. After stimulus presentation participants were asked whether the stimuli were the same or different. Participants had to select one of the named option buttons using the mouse which were presented left and right relative to the centre of the screen (randomised between subjects, consistent within subjects), after which they proceeded to the next trial. The two practise trials consisted of both a rhythm and melody trial (order random across subjects), and a single “same” and “different” trial (order random across subjects). After the practise trials the experimental trials started when participants were ready by pushing a start experiment button using the mouse.

Experimental trials were grouped in two blocks of 32 trials each, one rhythm and one melody block (order random across subjects). Half of the trials per block consisted of “same” trials, the remaining half of “different” trials. Balanced across these conditions, half of the trials contained a referent, the other half did not. In half of the “different” trials, the second stimulus either had a contour or a non-contour change. A target pattern was always presented as the first stimulus, and a specific target was presented during one trial for both “same” and “different.” Presentation order of these patterns was randomised across subjects, and manipulation size for the different trials was randomly selected during the experimental procedure. After these two experimental trial blocks, participants were asked the type of device used, what type of playback device used, whether they experienced difficulties and if they had any remarks or feedback.

### Statistics

All statistical analyses were conducted in R (r-project.org; v4.3.0) using RStudio (2036.06.0+421). Data were analysed using two separate approaches, the first using Signal Detection Theory (Macmillan and Creelman, 2004). Pooled parameters were calculated over subjects, i.e., sensitivity d' (reads as d-prime) and bias c, and transformed as per the “reminder design” (*d*' scaled by $\sqrt{2}$). To prevent zero values during computation of the *z-*score transform used in d‘ calculation from yielding infinity, such data was corrected by adding and subtracting 0.5 trials before the *z-*transform from their asymptotic counts. These parameters were subsequently analysed using generalised linear models (glm with gaussian family; “stats” package v4.3.0), after testing for normality using the Shapiro-Wilk Test (shapiro.test; “stats” package v4.3.0). Data were also analysed using the untransformed percentage correct measure, which were analysed using generalised linear mixed effects models with binomial logit link function (“lme4” package v1.1.33; Bates et al., 2014) with participant as random intercept. For both approaches, full models were created and reduced to the simplest significant models, where model significance was analysed using the “anova” function with Chi-square test (“stats” package v4.3.0). Pairwise contrasts were calculated using estimated marginal means (emmeans; “emmeans” package v1.8.6) with Šidák correction for multiple testing.

To test whether performance on melody and rhythm trials covaried, we analysed whether performance in rhythm trials was predictive of performance in melody trials using regression analyses. Individual participants' performance was assessed by calculating pooled *d*' for both rhythm and melody trials. Pooled *d*' was similarly calculated for each experimental factor (i.e., manipulation size and direction, contour impact, referent and framework) for both rhythm and melody to assess grouped participant performance over the experimental conditions. Correlation was assessed using linear regression (lm; “stats” package, v4.3.0) by including rhythm *d*' into the model as predictor. For all models, assumptions (e.g., homogeneity of variance and collinearity) were assessed using the “check_model” function (“performance” package v0.11.0). In all statistical tests, a *p-*value of < 0.05 was considered statistically significant.

## Results

### Experimental environment

Before addressing our research questions, we analysed the effect of participating online or in the lab environment. Both detection theory measures *d*' and *c* were found to be normally distributed (*p* = 0.801 and 0.182 respectively). Using these measures, we found no effect of participation environment on the sensitivity measure *d*' (β = 0.146 ± 0.105, *t* = 1.396, *p* = 0.165). Performance in the rhythm trails was found to be higher than melody trials (β = 0.331 ± 0.076, *t* = 4.382, *p* < 0.001), without interaction with participation environment (model rejection, *p* = 0.313; Supplementary Figure S2A). For the measure of bias c, we did find an effect of environment (β = 0.470 ± 0.175, *t* = 2.676, *p* < 0.01). Similar to *d*', we found a higher measure for rhythm trials as compared to melody trials (β = 1.008 ± 0.127, *t* = 7.940, *p* < 0.001) without an interaction effect with environment (model rejection, *p* = 0.439). These differences in bias mean that participants were more likely to respond “same” online than in the lab environment, without this response bias affecting the performance measure *d*'.

When using percentage correct as a performance measure we found the same results, i.e., no effect of environment (β = 0.143 ± 0.104, *t* = 1.374, *p* = 0.169) and a higher performance in rhythm trials (β = 0.358 ± 0.063, *t* = 5.690, *p* < 0.001; Supplementary Figure S2B) without an interaction (model rejection, *p* = 0.131). Based on these results we concluded that there was no relevant effect on performance between the two settings in which participants conducted this experiment, and hence all analyses below grouped these datasets into one.

### Rhythm

#### Signal detection theory d'

**Figure 2:**
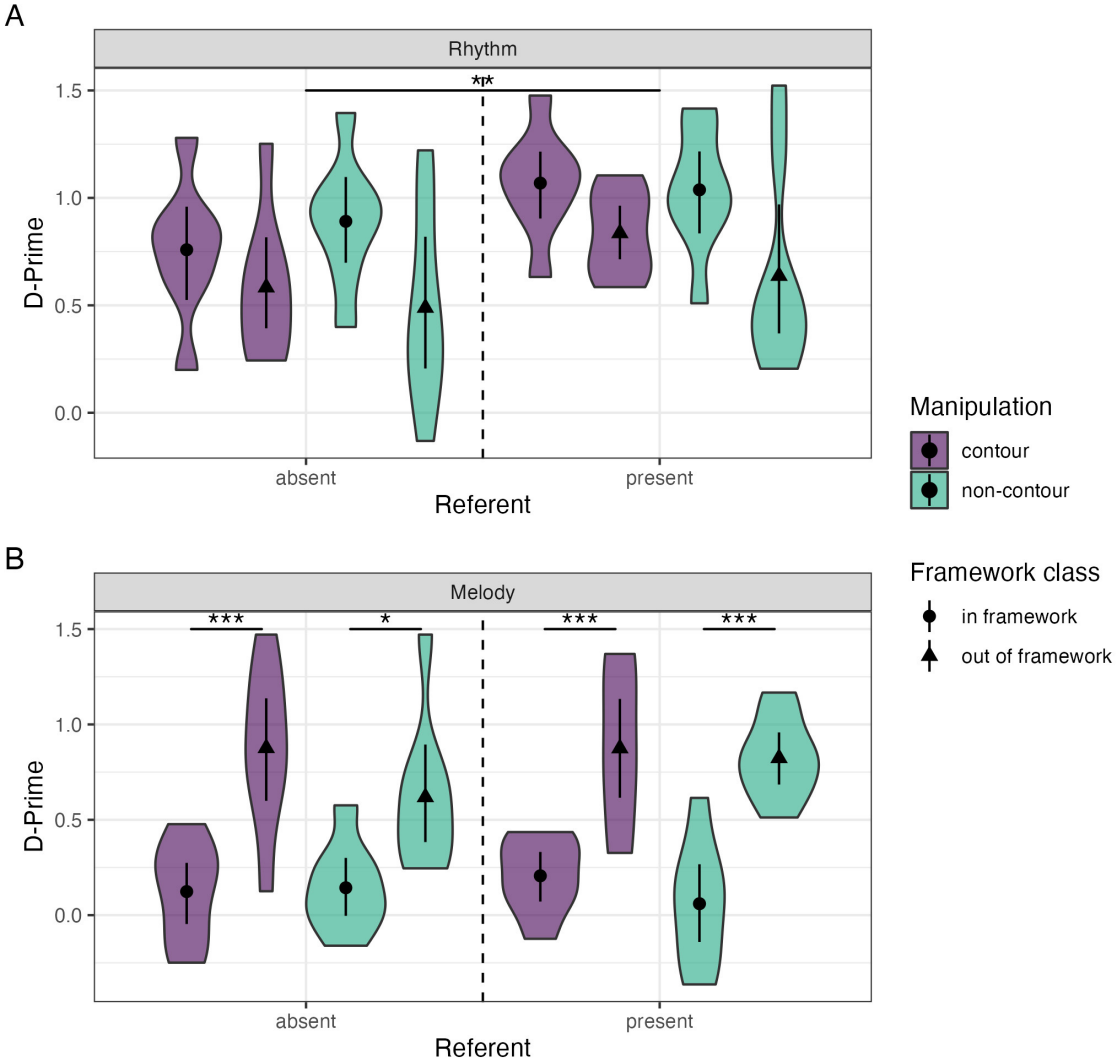
Distribution and mean (±95% confidence interval) of *d*' values for rhythm **(A)** and melody **(B)** trials for the main experimental conditions. Significance indicators; *** <0.001, ** <0.01, * <0.05.

No effects of musical training (model rejection, *p* = 0.514), or contour (model rejection, *p* = 0.534) were found for rhythm trials. Our final rhythm SDT *glm* showed a main effect of referent (β = 0.215 ± 0.073, *t* = 2.962, *p* < 0.01), without any interactions with other factors (model rejection, *p* = 0.756). There appears to be a main effect of framework (Figure 2A, β = −0.305 ± 0.083 *t* = −3.664, *p* < 0.001). We checked whether this could be explained by manipulation size (β = −*0*.305 ± 0.074, *t* = −4.122, *p* < 0.001), with both effects disappearing when including the marginally significant framework × manipulation size interaction in the model (*p* = 0.065). *Post-hoc* pairwise contrasts on this interaction showed an effect of framework for the small manipulations (in-framework—out-of-framework EMM = 0.436 ± 0.103, *t*= 4.220, *p* < 0.001) and an effect of the manipulation size for the out-of-framework condition (large–small manipulation EMM = 0.437 ± 0.102, *t*= 4.295, *p* < 0.001; Supplementary Figure S3A).

#### Percentage correct

**Figure 3:**
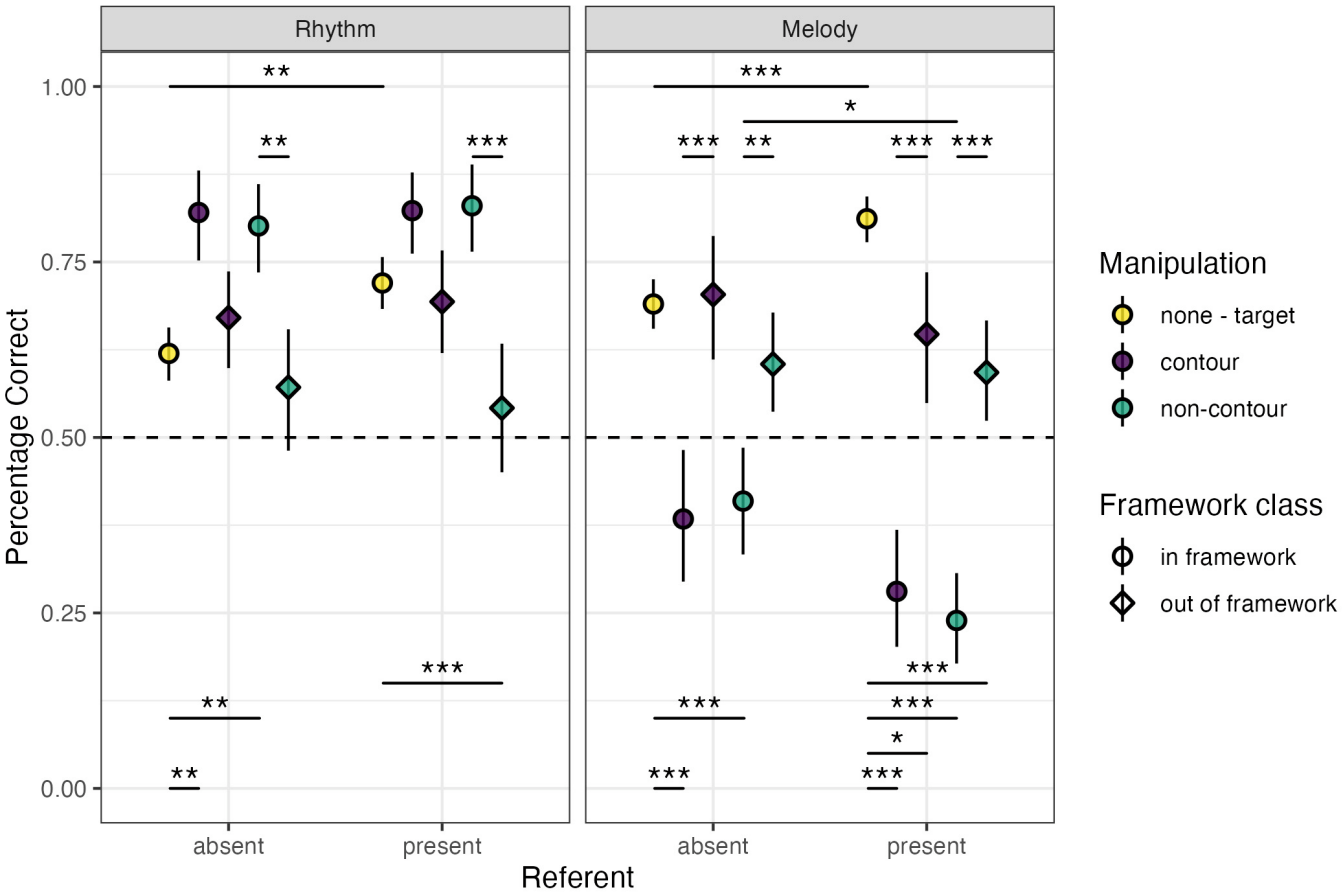
Mean (±95% confidence interval) percentage correct values for rhythm **(left)** and melody **(right)** trials for the main experimental conditions. Dashed-line marker at 50% (0.50) indicates chance-level performance. Significance indicators; *** <0.001, ** <0.01, * <0.05.

Once again, no main effect of musical training (model rejection, *p* = 0.332) was found for rhythm trials. Our final rhythm percentage correct *glmm* show a main effect for referent (β = 0.291 ± 0.095, *z* = 3.066, *p* < 0.01), without interactions with other factors (framework interaction model rejection, *p* = 0.657; contour interaction model rejection, *p* = 0.129). A significant contrast of referent was found only in the target trials (EMM = 0.509 ± 0.136, *z* = 3.748, *p* < 0.01), indicating higher performance with the referent present (Figure 3).

While a significant main effect difference of non-contour trials vs. contour trials was found in our final model (β = −*0*.306 ± 0.141, *z* = −2.172, *p* < 0.05), none of the contrasts between the contour conditions were found to be significant. However, a main effect of the target trials was found (β = −1.090 ± 0.171, *z* = −6.393, *p* < 0.001), which was reproduced in the in-framework no-referent contrasts (contour–target EMM = 1.062 ± 0.271, *p* < 0.01; non-contour–target EMM = 0.898 ± 0.233, *z* = 3.862, *p* < 0.01), but not the in-framework with-referent contrasts, or any of the out-of-framework contrasts with exception of the non-contour contrast (EMM = −*0*.897 ± 0.210, *z* = −4.280, *p* < 0.001; Figure 3). These results indicate a lower performance for the target (same) trials as compared to the different trials, especially in the in-framework trials. The increased performance in target trials with a referent present seems to diminish this difference to non-significance. Overall lower performance of the different trials with the out-of-framework manipulations also led to non-significant differences with the same trials, except for a lower performance in the non-contour with-referent condition.

There once again appears to be a main effect of framework (Figure 3, β = −*0*.766 ± 0.206 *z* = −3.715, *p* < 0.001). We similarly checked whether this could be explained by manipulation size (β = −*0*.738 ± 0.155, *z* = −4.764, *p* < 0.001), with both effects disappearing when including the significant framework × manipulation size interaction in the model (β = −*0*.717 ± 0.322, *z* = −2.229, *p* < 0.05). *Post-hoc* pairwise contrasts on this interaction showed an effect of framework for the small manipulation (in-framework–out-of-framework EMM = 1.058 ± 0.222, *z* = 4.775, *p* < 0.001), an effect of manipulation size as compared to the target in the in-framework-condition (large–target EMM = 0.917 ± 0.149, *z* = 6.170, *p* < 0.001; small–target EMM = 0.558 ± 0.206, *z* = 2.711, *p* = 0.052), and significant effects for all contrasts in the out-of-framework condition (large–small EMM = 1.109 ± 0.215, *t*= 5.162, *p* < 0.001; large–target EMM = 0.609 ± 0.198, *z* = 3.073, *p* < 0.05), with a large significant reduction in the small manipulation stimuli (small–target EMM = −*0*.500 ± 0.122, *z* = −4.113, *p* < 0.001; Supplementary Figure S3B). Same as the SDT analyses, these results indicate that the in the out-of-framework performance was significantly reduced mainly for the small manipulations.

### Melody

#### Signal detection theory d'

No main effects of referent (model rejection, *p* = 0.48), or contour (model rejection, *p* = 0.128) were found. Our final melody SDT *glm* indicated a main effect of framework (β = 0.663 ± 0.116, *t* = 5.706, *p* < 0.001; Figure 2B), and an interaction effect with musical training (β = 0.328 ± 0.134, *t* = 2.445, *p* < 0.05) without a main effect of training (β = 0.047 ± 0.095, *t* = 0.493, *p* = 0.624). In contrast to the rhythm trials, these effects persisted with manipulation size included in the model. Similar to the rhythm SDT analysis, for manipulation size an interaction was found with framework class (β = −*0*.324 ± 0.134, *t* = −2.412, *p* < 0.05), without a manipulation size main effect (β = 0.001 ± 0.095, *t* = 0.011, *p* = 0.991).

Contrasts of the framework × manipulation size interaction show a clear effect of framework for both small (EMM = −1.006 ± 0.19, *t* = −5.304, *p* < 0.001) and large manipulation sizes (EMM = −1.654 ± 0.19, *t* = −8.717, *p* < 0.001). Manipulation size was only found to have a significant effect for out-of-framework trials (large–small EMM = 0.645 ± 0.19, *t* = 3.400, *p* < 0.01), not the in-framework trials (EMM = −*0*.002 ± 0.19, *t* = −*0*.011, *p* = 1.000; see Supplementary Figure S3A). Similarly to the previous interaction, contrasts of the framework × training interaction indicate an effect of framework in both the group with (in-framework–out-of-framework EMM = −1.658 ± 0.19, *t* = −8.740, *p* < 0.001) and without musical training (EMM = −1.002 ± 0.19, *t* = −5.282, *p* < 0.001). Musical training was only found to increase performance in the out-of-framework trials (EMM = 0.749 ± 0.19, *t* = 3.951, *p* < 0.01) but not in the in-framework trials (EMM = 0.093 ± 0.19, *t* = 0.493, *p* = 0.999; see Supplementary Figure S4A). These contrasts above indicate lower performance due to small manipulations size, and increased performance due to musical training in the out-of-framework trials only.

#### Percentage correct

Our final melody percentage correct *glmm* indicated a main effect of referent (β = −*0*.641 ± 0.242, *z* = −2.654, *p* < 0.01), framework (β = 1.165 ± 0.236, *z* = 4.930, *p* < 0.001) and target trials (β = 1.121 ± 0.218, *z* = 5.136, *p* < 0.001), but not non-contour as compared to contour trials (β = −*0*.114 ± 0.184, z = −*0*.622 *p* = 0.534). A significant interaction of referent was found for target trials (β = 1.307 ± 0.280, *z* = 4.670, *p* < 0.001), and a marginal interaction with framework (β = 0.473 ± 0.259, *z* = 1.827, *p* = 0.068). No three-way interaction (model rejection, *p* = 0.334), or contour ^*^ framework interactions were found (model rejection, *p* = 0.216). Next to these, a significant interaction was found for manipulation size with framework (β = −*0*.680 ± 0.261, *z* = −2.601, *p* > 0.01), without a main effect of manipulation size (β = −*0*.063 ± 0.185, z = −*0*.340, *p* = 0.734). Finally, a significant interaction of musical training with framework was found (β = 0.550 ± 0.220, *z* = 2.493, *p* < 0.05), without main effect of training (β = 0.116 ± 0.128, *z* = 0.904, *p* = 0.366). No interactions were found for musical training with any of the other experimental predictors (model rejection, *p* = 0.965).

Results from our contrasts showed that the effect of the referent led to an increase in performance in the target trials (EMM = 0.665 ± 0.141, *z* = 4.713, *p* < 0.001) and a decrease in non-contour in-framework trials (EMM = −*0*.795 ± 0.242, *z* = −3.280, *p* < 0.05). All contour and non-contour contrasts were significantly lower compared to target trials for the in-framework stimuli (contour–target | no referent EMM = −1.291 ± 0.216, *z* = −5.968, *p* < 0.001; non-contour–target | no referent EMM = −1.276 ± 0.182, *z* = −6.472, *p* < 0.001; contour–target | with referent EMM = −2.420 ± 0.237, *z* = −10.226, *p* < 0.001; non-contour–target | with referent EMM = −2.636 ± 0.215, *z* = −12.285, *p* < 0.001), but none of the contour–non-contour in-framework contrasts were significant. For the out-of-framework stimuli, only the target stimuli with referent contrasts were significant (contour–target EMM = −*0*.785 ± 0.239, *z* = −3.280, *p* < 0.05; non-contour–target EMM = −1.06 ± 0.187, *z* = −5.656, *p* < 0.001). All framework contrasts (without target trials) showed significantly lower performance for the in-framework trials (no referent and contour EMM = −1.439 ± 0.293, *z* = −4.911, *p* < 0.001; no referent and non-contour EMM = −*0*.870 ± 0.225, *z* = −3.870, *p* < 0.01; with referent and contour EMM = −1.635 ± 0.300, *z* = −5.445, *p* < 0.001; with referent and non-contour EMM = −1.577 ± 0.240, *z* = −6.559, *p* < 0.001). See Figure 3 for the percentage correct results for referent, contour, and framework.

The lower performance in in-framework trials was maintained regardless of manipulation size (large manipulation EMM = −3.346 ± 0.378, *z* = −8.859, *p* < 0.001; small manipulation EMM = −1.872 ± 0.357, *z* = −5.247, *p* < 0.001) or musical training (without training EMM = −2.077 ± 0.299, *z* = −6.947, *p* < 0.001; with training EMM = −3.142 ± 0.373, *z* = −8.806, *p* < 0.001). The effect of manipulation size was once again found to be limited to the out-of-framework stimuli (large–small EMM = 1.536 ± 0.359, *z* = 4.274, *p* < 0.001; Supplementary Figure S3B). Similarly, only in out-of-framework trials did participants with musical training display significantly higher performance (EMM = 0.661 ± 0.194, *z* = 3.412, *p* < 0.01; Supplementary Figure S4B).

### Melody and rhythm performance compared

Regression analysis of individual participant performance in melody trials indicated that performance in rhythm trials was non-predictive (β = −*0*.039 ± 0.086, *t* = −*0*.461, *p* = 0.646). Significant predictors of individual participant performance in melody trials were found to be musical training (β = 0.230 ± 0.090, *t* = 2.566, *p* < 0.05), and the type of framework trial (β = 0.601 ± 0.086, *t* = 6.968, *p* < 0.001), without an interaction effect (model rejection *p* = 0.231), with the two factors explaining 27.37% of between participant variance in melody trials (adjusted *R*^2^; Supplementary Figure S5).

**Figure 4:**
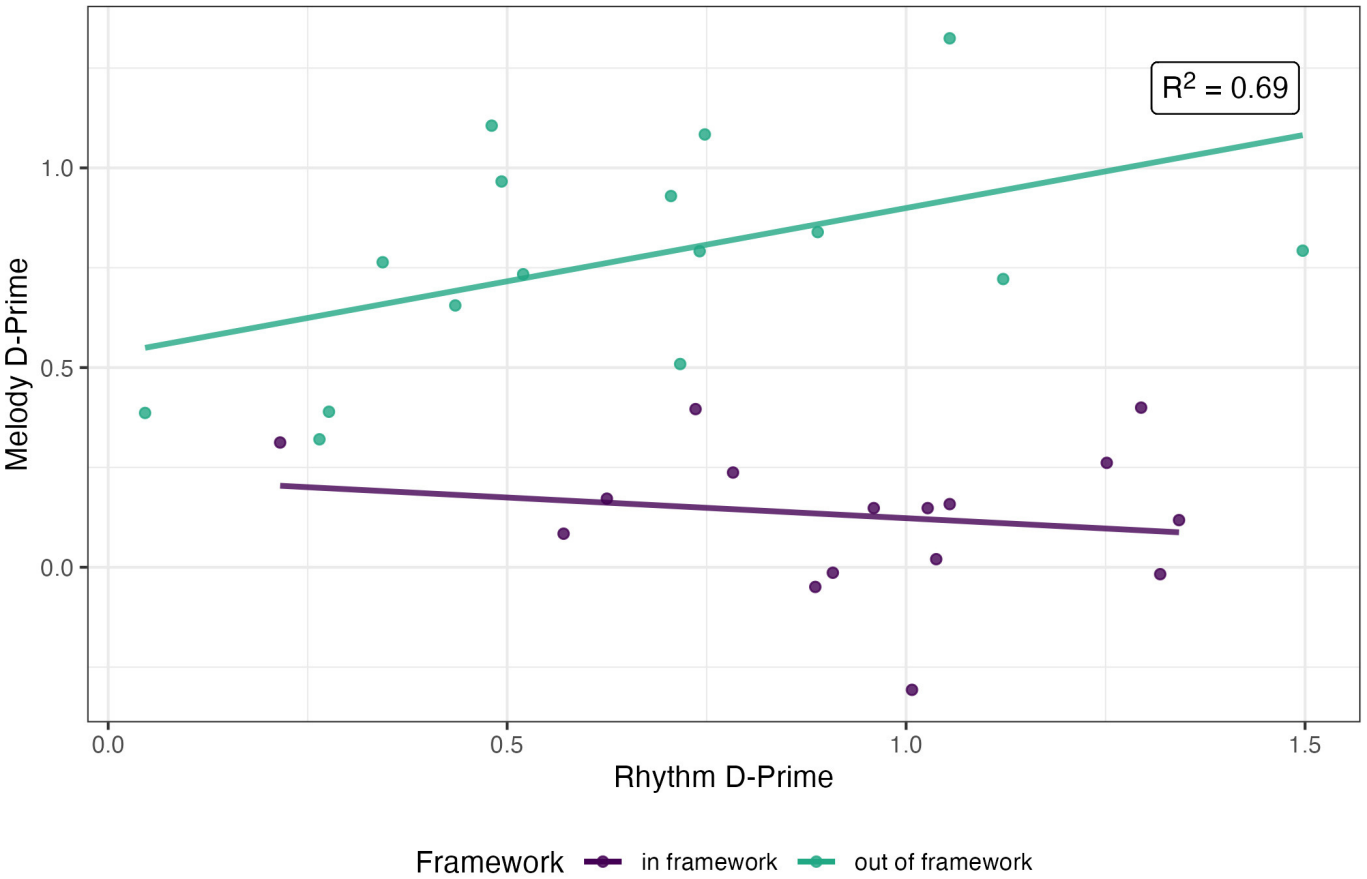
Regression of grouped performance across experimental conditions (individual points) for melody trials in relation to performance for rhythm trials.

Regression analysis of grouped participant performance in melody trials indicated that performance in rhythm trials was marginally predictive in interaction with the type of framework trials (β = 0.471 ± 0.243, *t* = 1.941, *p* = 0.062), without a main effect of either predictor (rhythm performance β = −*0*.106 ± 0.187, *t* = −*0*.553, *p* < 0.585; framework β = 0.306 ± 0.216, *t* = 1.414, *p* < 0.168). The estimated slope of performance in melody trials as a function of performance in rhythm trials, i.e., the covariance, was found to be 0.367 (±0.154, *t* = 2.379, *p* > 0.05) for out-of-framework stimuli. This model could explain 69.38% of the variance in melody performance between experimental conditions (adjusted *R*^2^, Figure 4).

## Discussion

In this experiment we used a same/different paradigm test the hypothesis that relative pitch and relative rhythmic perception rely upon some shared cognitive capabilities. Our results showed that a rhythmic or tonal referent (bass-drum beat or in-key drone note, respectively) increased performance in both rhythm and melody trials, for the target stimuli in particular. Next to this, the referent seemed to further lower the already below chance (incorrect) response for in-framework (i.e., in-key) melody stimuli. This suggests the referent reinforced the melodic percept participants were experiencing, thus solidifying the conclusion of the in-framework stimulus being the same as the target stimulus. Surprisingly, a change in contour did not affect performance, and thus did not appear to influence perception in either rhythm or melody trials. Finally, though no individualised correlation was found for within-participant performance, on a group level a clear correlation was found between rhythm and melody performance for out-of-framework (i.e., out-of-key and lower metricality) stimuli only. Although these results differ from our earlier predictions, the effects of most manipulations and general performance covaried between the rhythm and melody trials and are thus congruent with the underlying hypothesis of shared relative perception.

As predicted, we found an effect of referent on perception in both rhythm and melody trials. Specifically, we predicted the presence of the referent would make the task easier for participants, which we found for the target (i.e., “same”) trials for both rhythm and melody. The increase in same-trial performance in the rhythm conditions is responsible for the main effect of referent found in the Signal Detection Theory analysis, as all results in the analysis are relative to performance in the default condition which is the target trial. One could argue that judging two stimuli as being the same is more difficult than judging them as being different, as the former requires every interval to be judged as identical, while the latter only requires detection of one different interval. Regardless, we might have expected an effect of referent on the “different” trials because we predicted this task to be easier due to the additional information, similar to the “same” trials.

For the melody condition, we also found a positive effect of the referent on the target trials, to our knowledge representing the first experimental evidence of an effect of a melodic drone on melody perception. Additionally, the already below chance level performance of different in-framework (in-key) melody trials was reduced even further by the addition of a referent. This suggests that participants did not perceive the in-framework melody as being different from the target stimuli, and the presence of the referent further reinforced this conclusion. Interestingly, in all conditions the referent only seemed to affect trials where participants mainly gave the “same” response, which suggests the referent might not be beneficial for difference detection *per se*. If we assume the “same” response to be the default that is disproven by the detection of a different interval, then the referent might be responsible for increasing participant certainty regarding the lack of any difference. The type of referent used in this experiment aligns with the frameworks used, either the metre in rhythm stimuli or the tonic of the key in melody stimuli. We predict that a reinforcing effect of a referent is contingent on whether the referent aligns with the rest of the relevant domain, other musical features and the perceiver's musical exposure. As such, a referent that is not aligned with its domain's features might interfere with perception, and as such could act as a distraction.

Although in general, manipulations had similar effects for rhythm and melody, for the framework manipulation this was not the case. Specifically, although both metre and melodic key create a grid of expectations for likelihood of following events, deviations adhering to rhythmic expectations were successfully detected in rhythm trials. In contrast, in-key changes to melodic stimuli were not detected, and thus scored as being the same as the target, an effect further amplified by the presence of the melodic drone referent. This suggests that even if relative perception is shared, there are also differences and specialisations specific to rhythm and melody. Interestingly, for both rhythm and melody out-of-framework stimuli, not the in-framework stimuli, small manipulations appeared to be harder to detect than the large manipulations. Despite this measurable decrease in performance, the small manipulations used here are much larger than the just noticeable difference for both rhythm (< 10 ms for our IOIs, Drake and Botte, 1993; Friberg and Sundberg, 1995) and melody (~20 cents for major scales, Lynch et al., 1991). Perhaps the perception for the out-of-framework stimuli is more reliant on the general relative feature perception than for the in-framework expectations specific for both domains, thus leading to more errors for the harder to detect smaller deviation. The fact that performance for small manipulation in-framework rhythm stimuli was unaffected, while our small manipulations have a higher impact on the LHL-FR syncopation score and are therefore fundamentally more likely to be classified as out-of-framework, also aligns with this potential explanation.

Finally, the effect of musical training on performance was limited to the out-of-framework melody stimuli. While it is likely that exposure to in-framework musical structures is common for all participants and musical training makes it more probable to be to exposed alternative implementations, we would expect the same to be true for both rhythm and melody. Since musically trained individuals are better at detecting key and out-of-key accompaniment (Wolpert, 1990), our untrained participants may not have found this cue as salient and therefore had greater difficulty detecting the difference between the stimulus and the target. Of our musically trained participants, only 4 had experienced dance training exclusively, while 16 had exclusively instrument training and 6 participants experienced both. Perhaps this bias towards instrumentalists led to the melody-skewed effects of musical training seen in our data.

In contrast to our prediction, we did not find an effect of contour in either the rhythm or melody trials. Contour has long since been well-established as a relevant factor in melody perception (Dowling and Fujitani, 1971), with even young infants displaying sensitivity to contour (Trehub et al., 1984). In addition to its role in melody perception, contour has also been suggested to be play a role in loudness and timbre perception (McDermott et al., 2008), and recently also been found to have an effect in rhythm perception (Schmuckler and Moranis, 2023). Many different stimulus designs have been used across these different experiments, which could explain our contour null result. Previous experiments using short stimuli, i.e., 4 or 5 intervals, might have made the short-term memory requirements of the task substantially easier compared to our considerably longer stimuli (e.g., Dowling and Fujitani, 1971; McDermott et al., 2008; Trehub et al., 1984). Similarly, experiments where contour was changed over many intervals may have made the perceptual demands of those tasks less challenging as compared to our 2 interval changes without impacting the rest of the contour (e.g., Schmuckler and Moranis, 2023; Weiss and Peretz, 2024). Finally, contour has been shown to create top–down expectations on what interval is predicted to follow (Ishida and Nittono, 2024). As such, experiments with stimuli using more structured patterns, (e.g., ascending, descending or arch-shaped), or even familiar melodies, are likely to make perception less demanding compared to our randomly generated stimuli (e.g., Dowling and Fujitani, 1971; Ishida and Nittono, 2024). Regardless of the possible cause for a lack of an effect of contour in our study, the null effects found here were consistent between rhythm and melody conditions.

Although we hypothesise that shared perceptual mechanisms may be used in rhythm and melody perception, it is well-established that they are also functionally and anatomically separate from each other. Previous studies show that brain lesions can impair capabilities in one domain without affecting the other (e.g., Di Pietro et al., 2004; Peretz, 1990). In general, melody seems to be predominantly processed by the right hemisphere, while rhythm is processed bilaterally including involvement of the (pre)motor cortex, basal ganglia and cerebellum (De Angelis et al., 2018; Peretz and Zatorre, 2005). Previous data also indicate a separation of the domains for working memory (Jerde et al., 2011). Despite these differences in neural substrates, a study looking at musicians with absolute pitch and those without, i.e., with only relative pitch, found higher activation for relative pitch for the pre-supplementary motor area (Leipold et al., 2019), an area previously associated with rhythm tasks (Bengtsson et al., 2009; Grahn, 2012). Next to this, the differences commonly found between rhythm and melody in terms of their neural substrates seem to develop over ontogeny and are less pronounced in younger children age 5–7 (Overy et al., 2004). This suggests that rhythm and melody might be dependent on similar perceptual processes, and that the differences seen in rhythm and melody perception and neural substrates develop over ontogeny with musical exposure and training. This is further supported by research showing that tasks involving either rhythm or pitch perception tend to recruit many similar brain areas, and thus display a high degree of similarity (Griffiths et al., 1999; Siman-Tov et al., 2022), including the involvement of the cerebellum for pitch tasks. So, despite rhythm and melody processing being partially separated from each other, there does seem to be some overlap between the two domains, including across their neural substrates.

Even though our results are congruent with the hypothesis that rhythm and melody share relative perception, there are some limitations to our experimental design and results. Firstly, we did not find that performance in rhythm and melody trials covaried within individual participants. Due to the design of this experiment and the many stimuli involved, participants rarely experienced the exact same set of stimuli as each other, despite receiving the same experimental conditions, leading to high variance in participant performance. Nonetheless, if rhythm and melody perception are simply unrelated, an alternative explanation for the strong correlation of the grouped regression analysis found here would be required, as it can't be simply explained by musical experience and/or training. Now that we have established it is fruitful to compare rhythm and melody perception in parallel, future work investigating the mechanisms underlying this correlation could shed light on its causal nature and potential neural basis.

As our study was a first step to explore shared relative perception between rhythm and melody, we only looked at a limited set of possible frameworks. For melody stimuli we used the major pentatonic scale, while many alternatives could have been chosen. This includes for example: minor scales, blues scales, heptatonic scales (e.g., major diatonic) or any of the numerous non-western scales. Interestingly, two specific manipulations were scored as being out-of-framework in our analyses that would fit within the major heptatonic (i.e., diatonic) scale (+/– 3 semitones for the major second to produce the perfect fourth and major seventh, respectively). However, we consider the potential in-framework effects of these specific manipulations to be of low impact on our results and conclusions, as the out-of-framework melody performance remains clearly different from the in-framework melody trials and is comparable to the out-of-framework rhythm performance. Similarly, we constructed our rhythmic stimuli using a 4/4 metre, the most common western metre. In the case of the rhythms without referent, this metre was not enforced, so it is possible that participants interpreted these as having a different metre (or perhaps none). Despite our usage of these western musical frameworks, we tried to avoid WEIRD sampling regarding our online participants (see Table 1; Henrich et al., 2010), although all of our participants did have a working computer with internet connection available, and thus likely had at least some western music exposure.

Many other principles of organisation are also important in both rhythm and melody, like (perceptual) grouping, repetition of elements, and the formation of simple patterns (Krumhansl, 2000). Such musical principles are weak or absent in our randomly generated stimuli, and this might have affected our results, as the goal of the randomised stimuli was to control for such factors. Despite these limitations, we consider the results to be consistent with the hypothesis that rhythm and melody exhibit at least some overlap in relative perception mechanisms. What the actual mechanism underlying the correlation described here is, is unfortunately not within the scope of current study due to the design of the experiment. Neither do we argue whether low-level acoustic features, or high-level cognitive processes, are the defining component of the perceptual mechanism. We do hypothesise the same process being used to perceive the relative information encoded in both the temporal and spectral information that represent rhythm and melody, respectively. Potential candidates could for example be rooted in predictive coding (Huang and Rao, 2011; Kölsch et al., 2019; Rao and Ballard, 1999), and/or a phase hierarchy approach (Goswami, 2019), which when applied to amplitude modulation appears predictive of rhythmic processes and perception (e.g., Chang et al., 2024).

Many different experimental extensions could provide further insights into the nature of the relationship between rhythm and melody perception in the future. One interesting question would be whether the reduced imitation capabilities of absolute features in individual with ASD (Wang et al., 2021) translates to differences in relative perception. Functional magnetic resonance imaging (fMRI) studies using this population, or with congenital amusics, could provide insights into the neural substrates involved in absolute and relative feature perception, since we would predict different neural activation patterns underlying these differences in perceptual capabilities. In a similar vein, most fMRI studies to date focus on the difference in neural substrates between rhythm and melody and their specialisations (e.g., Bengtsson and Ullen, 2006; Kasdan et al., 2022; Thaut et al., 2014). More studies focussing on overlap between these two key musical domains could provide fundamental insights into their underlying mechanisms, possibly utilising the contrast between culturally emergent domain-specific expectations compared to more general perceptual mechanisms.

Finally, another potentially interesting alternative avenue of research would be comparative studies of the relative pitch capabilities of non-human animal species known to be capable of rhythmically entraining to music. Entrainment to a musical beat is dependent on detecting the beat, and perhaps metre, and most likely involves relative perception. Of particular interest would be the relative pitch capabilities of parrots. Not only are they the only group of animals consistently found to spontaneously engage in beat perception and synchronisation (Patel, 2021; Patel et al., 2009; Schachner et al., 2009), but relative pitch might also be beneficial for their vocal imitation capabilities (Moore, 2004). This potential link is further amplified by the suggestion that parrot beat entrainment could be linked to vocal pitch control related to their vocal learning circuitry (Patel, 2024). Thus far only the budgerigar has been studied in controlled settings in this regard, and no evidence was found for octave equivalence (Wagner et al., 2019) in contrast to for example rats (Wagner et al., 2024), and a lack of preference was found for consonance in chords (Wagner et al., 2020). One previous study using a single African Grey parrot (*Psittacus erithacus*) did find evidence supportive of relative pitch perception, documenting untrained and flexible vocal transpositions to other octaves than those prompted (Bottoni et al., 2003). These relative pitch capabilities align with rhythmic capabilities shown thus far in parrots, which document limited precision and flexibility in budgerigars as opposed to some other parrots (for example cockatoos and African Greys; Hasegawa et al., 2011; Schachner et al., 2009; Seki and Tomyta, 2019). Thus, more data is needed to investigate if parrot relative pitch perception relates to the relative rhythmic skills.

In conclusion, this experiment sought to investigate whether rhythm and melody share cognitive capabilities underlying relative perception and found evidence congruent with this hypothesis in a non-WEIRD population, allowing for generalisability. We found shared effects of different manipulations, alongside domain specific differences, and a corelation in performance across the two domains. This included the perceptually reinforcing effect of adding relative information in the form of a referent, which is also the first experimental evidence of an effect of a melodic drone on melody perception. These results therefore open the door to future more detailed exploration of relative perception in these two crucial aspects of human music.

## Data Availability

The datasets presented in this study can be found in online repositories. The names of the repository/repositories and accession number(s) can be found in the article/Supplementary material. The collection containing stimulus generation notebook, experimental stimuli, experiment, raw data and R statistics markdown for this study can be found in PHAIDRA.
