# Supplementary material for: Evidence for a shared cognitive mechanism underlying relative rhythmic and melodic perception
Source: Front Psychol. 2025 Jan 15;15:1512262. doi: 10.3389/fpsyg.2024.1512262 (PMC11774853; doi:10.3389/fpsyg.2024.1512262)
Supplement: Supplementary file 1 [file Data_Sheet_1.pdf]

Supplementary Material

Evidence for a shared cognitive mechanism underlying relative rhythmic and melodic perception

Jeroen van der Aa[1*], W. Tecumseh Fitch[1]

[1] Department of Behavioral and Cognitive Biology, Vienna CogSciHub, University of Vienna, Vienna, Austria

*** Correspondence:**
Jeroen van der Aa and W. Tecumseh Fitch
jeroen.van.der.aa@univie.ac.at and tecumseh.fitch@univie.ac.at

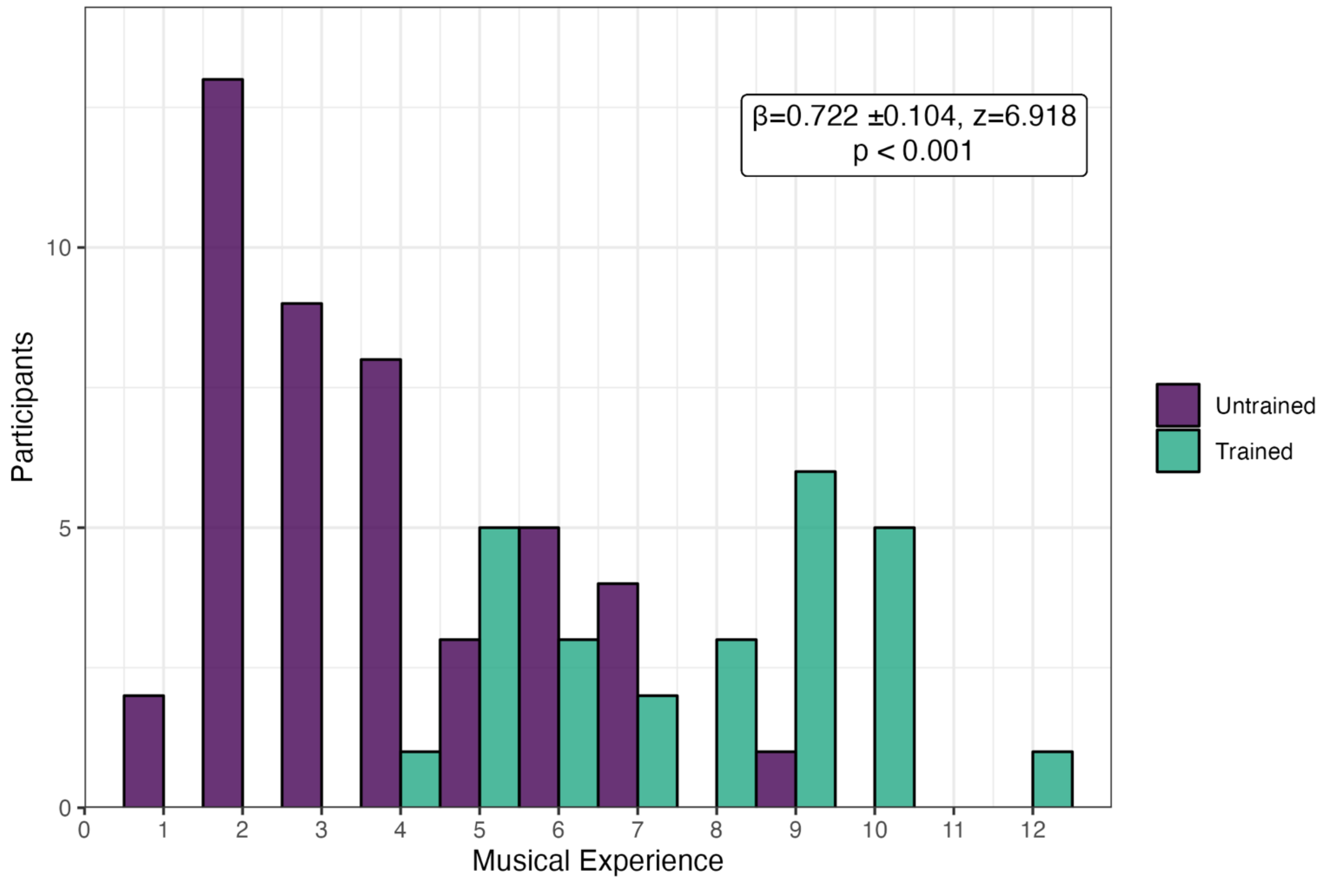

Figure S1. Distribution and statistical significance of participant musical experience in relation to musical experience split as used in later analyses.

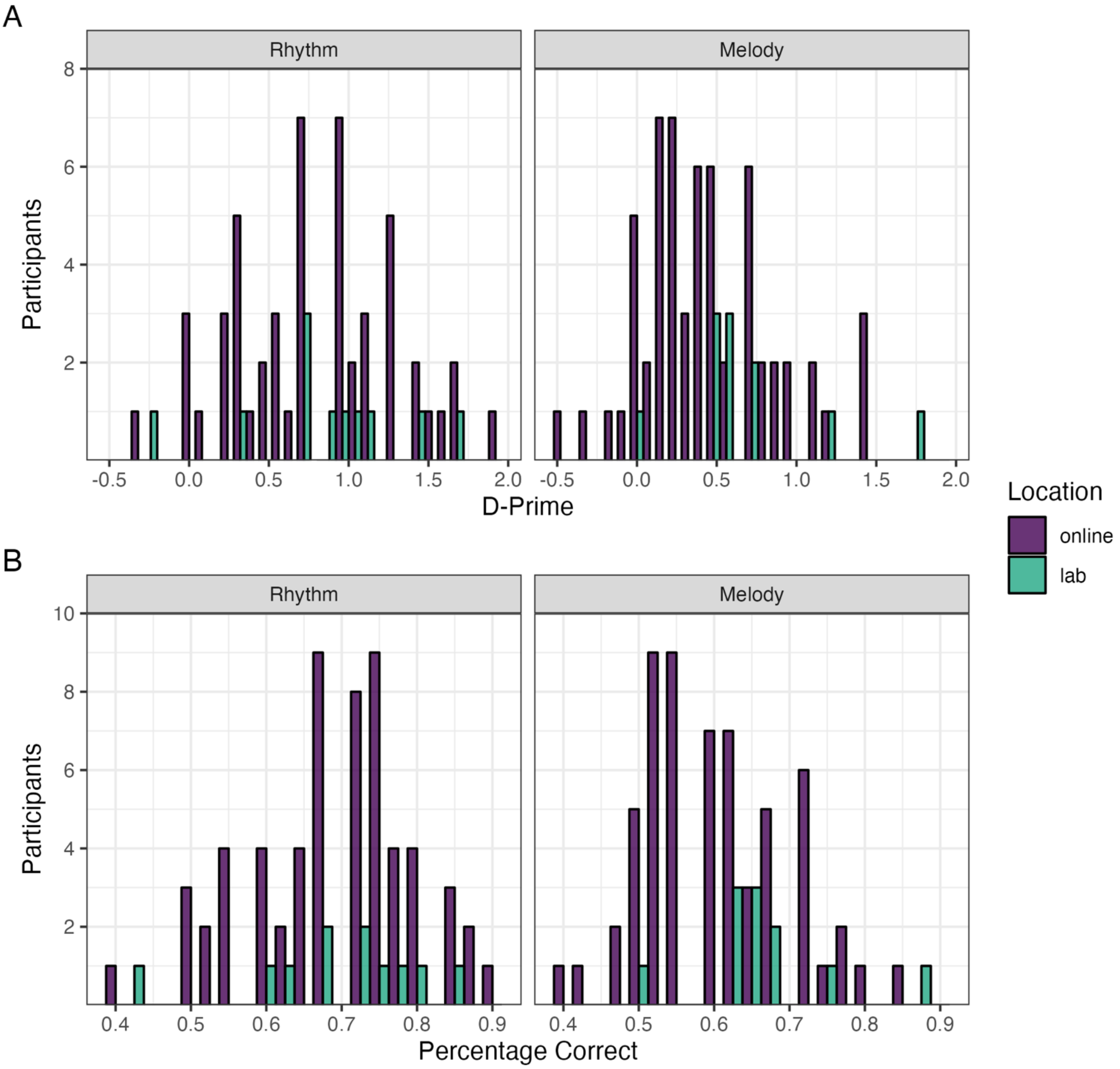

Figure S2. Participant performance for rhythm (left) and melody (right) trials for the different experimental environments. A) Performance using d’ as performance measure. B) Percentage correct as performance measure.

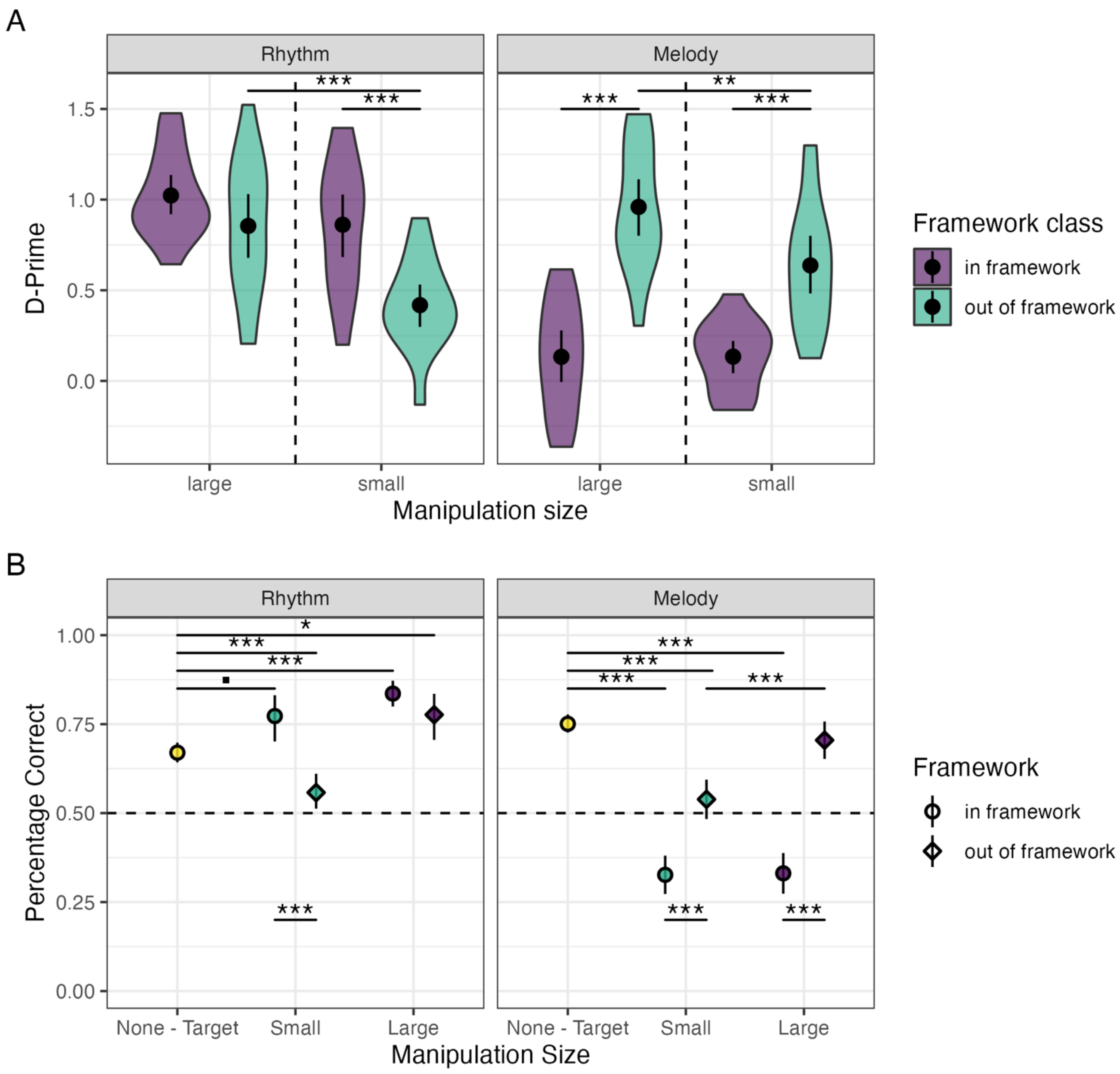

Figure S3. Participant performance for rhythm (left) and melody (right) trials for different manipulation sizes. Significance indicators; *** < 0.001, ** < 0.01, * < 0.05, ■ < 0.10.
A) Distribution and mean (± 95% confidence interval) d’ as performance measure. B) Mean (± 95% confidence interval) percentage correct as performance measure with marker at 50% to indicate chance-level performance. Colours do not indicate framework class, yellow = same (i.e. target) trial performance, other colours = different trials.

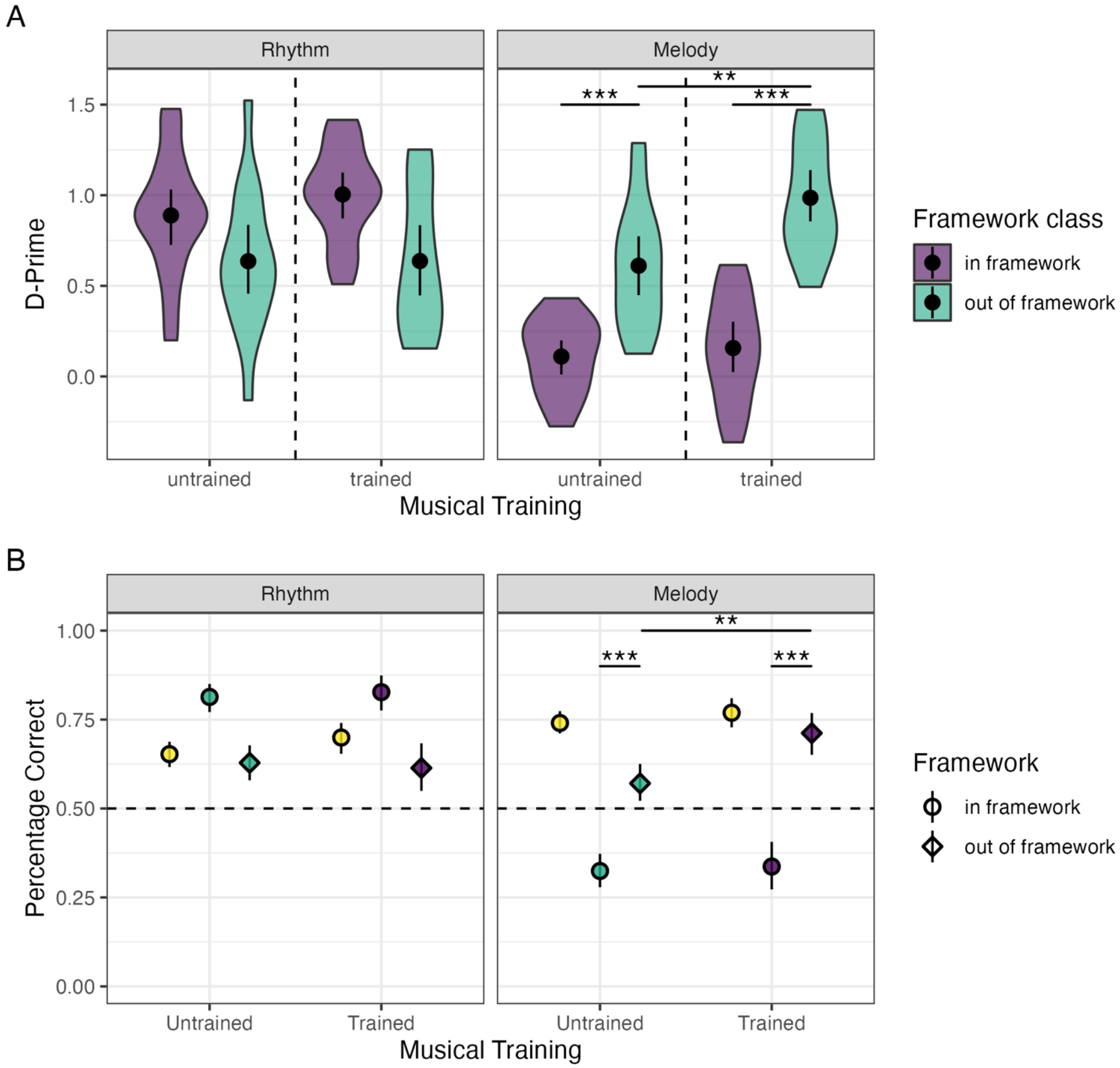

Figure S4. Participant performance for rhythm (left) and melody (right) trials for different preexisting musical training. Significance indicators; *** < 0.001, ** < 0.01.

A) Distribution and mean (± 95% confidence interval) d' as performance measure. B) Mean (± 95% confidence interval) percentage correct as performance measure with marker at 50% to indicate chance-level performance. Colours do not indicate framework class, yellow = same (i.e. target) trial performance, other colours = different trials.

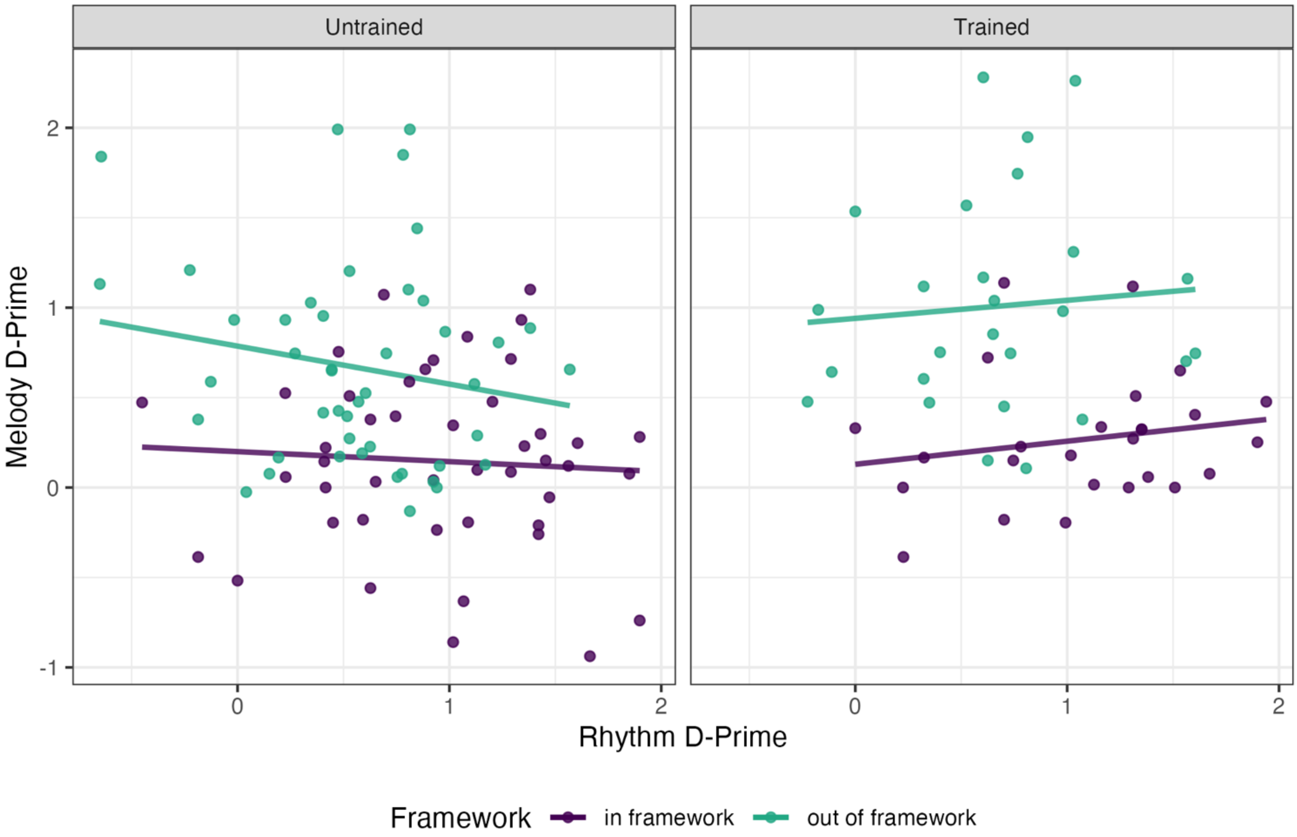

Figure S5. Regression of individual performance across all trials (individual points) for melody trials in relation to performance for rhythm trials.
